# Slow Down: Behavioural and Physiological Effects of Reducing Eating Rate

**DOI:** 10.3390/nu11010050

**Published:** 2018-12-27

**Authors:** Katherine Hawton, Danielle Ferriday, Peter Rogers, Paula Toner, Jonathan Brooks, Jeffrey Holly, Kalina Biernacka, Julian Hamilton-Shield, Elanor Hinton

**Affiliations:** 1 National Institute for Health Research Bristol Biomedical Research Centre, University Hospitals Bristol NHS Foundation Trust and University of Bristol, Bristol BS8 1TU, UK; peter.rogers@bristol.ac.uk (P.R.); pt13294@my.bristol.ac.uk (P.T.); j.p.h.shield@bristol.ac.uk (J.H.-S.); elanor.hinton@bristol.ac.uk (E.H.); 2 Nutrition and Behaviour Unit, School of Psychological Science, University of Bristol, 12A Priory Rd, Bristol BS8 1TU, UK; danielle.ferriday@bristol.ac.uk; 3 Clinical Research and Imaging Centre, University of Bristol, 60 St Michael’s Hill, Bristol BS2 8DX, UK; jon.brooks@bristol.ac.uk; 4 School of Experimental Psychology, University of Bristol, 12A Priory Rd, Bristol BS8 1TU, UK; 5 IGFs and Metabolic Endocrinology, University of Bristol, Second Floor, Learning and Research, Southmead Hospital, Westbury-on-Trym, Bristol BS10 5NB, UK; jeff.holly@bristol.ac.uk (J.H.); mdxkz@bristol.ac.uk (K.B.)

**Keywords:** eating rate, satiety, functional magnetic resonance imaging (fMRI), memory for recent eating, appetite hormones, meal enjoyment

## Abstract

Slowing eating rate appears to be an effective strategy for reducing food intake. This feasibility study investigated the effect of eating rate on post-meal responses using functional magnetic resonance imaging (fMRI), plasma gastrointestinal hormone concentrations, appetite ratings, memory for recent eating, and snack consumption. Twenty-one participants (mean age 23 years with healthy body mass index) were randomly assigned to consume a 600 kcal meal at either a “normal” or “slow” rate (6 vs. 24 min). Immediately afterwards, participants rated meal enjoyment and satisfaction. FMRI was performed 2-h post-meal during a memory task about the meal. Appetite, peptide YY, and ghrelin were measured at baseline and every 30 min for 3 h. Participants were given an ad-libitum snack three hours post-meal. Results were reported as effect sizes (Cohen’s d) due to the feasibility sample size. The normal rate group found the meal more enjoyable (effect size = 0.5) and satisfying (effect size = 0.6). Two hours post-meal, the slow rate group reported greater fullness (effect size = 0.7) and more accurate portion size memory (effect sizes = 0.4), with a linear relationship between time taken to make portion size decisions and the BOLD response in satiety and reward brain regions. Ghrelin suppression post-meal was greater in the slow rate group (effect size = 0.8). Three hours post-meal, the slow rate group consumed on average 25% less energy from snacks (effect size = 0.5). These data offer novel insights about mechanisms underlying how eating rate affects food intake and have implications for the design of effective weight-management interventions.

## 1. Introduction

Obesity is a major public health issue with approximately 60% of adults and nearly 30% of children aged 2–15 years in the UK being overweight or obese [1]. There is a paucity of strategies proven to be effective for preventing, or treating, overweight or obesity. Fast eating is associated with excess body weight [2] and reducing rate of eating appears to reduce energy intake [3]. These systematic reviews have highlighted that further work is needed to ascertain the mechanism underlying effects of eating rate on weight control and whether eating rate influences self-reported appetite [4]. For example, a recent study found that participants who consumed a test meal more slowly reported a greater increase in fullness, yet with reduced enjoyment and satisfaction from the meal [5].

Research investigating the physiological basis of the effect of eating rate on satiety has yielded inconsistent results for gastrointestinal (GI) hormone responses. One study utilising a cross-over design (*N* = 17) found greater post-prandial peptide YY (PYY) response after slowing eating rate (30 min vs. 5 min meal) however no effect was seen on ghrelin suppression [6]. Similar findings were elicited by another study examining the effects of eating rate and eating density on GI hormone responses (*N* = 20), where slow eating rate (20 g/min vs. 80 g/min) led to greater PYY response but no effect on ghrelin suppression [7]. By contrast, one cross-over study (*N* = 25), found no effect of eating rate by comparing 7 min, 14 min and 28 min meals on PYY release post-prandially [8]. A study of overweight adolescents (*N* = 27) utilising a Mandometer (device providing real-time feedback about consumption of meal by weight of plate) to slow eating rate vs. control, found slowing eating rate led to increased PYY response and greater ghrelin suppression post-prandially [9]. Thus, there is no current consensus regarding the effect of eating rate on satiety hormone response and further research is needed.

Other work has explored whether the mechanism underlying differences in food intake following different eating rates may relate to enhancing or disrupting memory for recent eating episodes [10,11]. There is accumulating evidence that memory for recent eating plays a pivotal role in the control of energy intake [12,13,14]. A recent study (*N* = 40) found that participants who consumed a meal more slowly remembered eating a larger portion but eating more slowly was not found to affect self-reported memory vividness [10], as was found in another study [11]. Memory for recent eating is assumed to involve brain regions classically associated with memory, such as the hippocampus [15,16]. More recent functional magnetic resonance imaging (fMRI) studies utilising a “What, Where, When” paradigm of episodic memory [17,18] suggest that, in addition to the hippocampus and other medial temporal lobe regions, frontal and parietal brain regions are also important for memory-related processes.

The objectives of this feasibility study were two-fold: first, to assess the feasibility of conducting a further, full-powered study combining a range of different measures (appetite ratings, satiety hormones, fMRI and subsequent consumption) to assess the effect of manipulating eating rate on these measures relating to energy intake. Feasibility outcomes were (i) combining novel and technically challenging measures (how many full data sets were obtained), (ii) a novel fMRI task (were the participants able to perform the task), (iii) ability to measure imaging signal in the brain regions of interest using fMRI, (iv) the practicality and acceptability of the blood sampling protocol (number of samples obtained) and (v) whether participants were aware of the aim of the study. Neuroimaging, a novel memory for recent eating task and GI hormones measurements were combined to ascertain whether top-down cognitive mechanisms or bottom-up physiological mechanisms are driving the effect of slowing eating rate. We developed a new “what, where, when” task to assess for memory for recent eating to be utilised during fMRI, as no such paradigm was previously available. Secondary objectives were to provide preliminary data using measures of appetite, hormones, fMRI and ad-libitum consumption to gain a greater understanding of the mechanisms underlying the effect of eating rate on energy intake.

To our knowledge, this is the first study to apply neuroimaging to investigate the effects of eating rate. The extensive fMRI literature on the satiating effects of food and food cues predicts a “network” of brain regions whose response might be influenced by eating rate, including insula [19] and orbitofrontal cortex (OFC) [20], in addition to subcortical reward regions [21,22,23]. We predicted that slowing eating rate would lead to greater signal change during a memory for recent eating task in memory-, satiety- and reward-responsive brain areas, including hippocampal, frontal and parietal regions. We also hypothesised that experimentally slowing eating rate would lead to reduced enjoyment and satisfaction, a greater feeling of fullness post-meal and improved memory of the meal. In relation to GI hormones, we hypothesised that slowing eating rate would lead to increased ghrelin suppression and increased PYY secretion post-meal. We predicted that the combined effect of these mechanisms would lead to a reduced subsequent food intake at an *ad-libitum* snack meal.

## 2. Materials and Methods

### 2.1. Participants

Twenty-one participants aged between 18–35 years of healthy BMI (18.5–25.0 kg/m^2^) were recruited using posters and an online advert. Participants were asked to complete an online questionnaire to check eligibility which also included the Dutch Eating Behaviour Questionnaire (DEBQ) [24]. Ethical approval was granted by the Faculty of Science Human Research Ethics Committee, University of Bristol.

### 2.2. Eligibility

To be eligible, the body mass index (BMI) of participants had to be within the normal range (18.5 kg/m^2^–25 kg/m^2^). Any volunteers who had a history of eating disorders, neurological disorders or traumatic brain injury, psychiatric disorders or diabetes were excluded. Participants who were on a diet to control weight, were pregnant or had food allergies, were taking any medication that might influence appetite or who smoked more than five cigarettes per day were also excluded. It was also a requirement for participants to be fluent in English. To ensure safety in the MRI scanner, further contraindications included metal implants and any tattoos with metallic ink, which were screened for using the standardised Clinical Research Imaging Centre (CRIC Bristol) screening form. A signed consent form was completed by each participant prior the participating in the study.

### 2.3. Randomisation

Participants were allocated on the basis of age, gender, BMI and DEBQ restraint scores to either a “normal” or “slow” eating rate group. To balance the groups on these factors, a minimisation method proposed by Pocock & Simon [25] was employed. This adaptive randomization technique is designed to reduce any imbalance between the experimental groups in the distribution of scores over several factors [26]. A 4:1 element of chance was incorporated and automated using Microsoft Excel so that allocation to groups was pseudo-random [27]. Eleven participants were randomised to the “normal” group and 10 participants were randomised to the “slow” group.

### 2.4. Measures

Computerised visual analogue scales (VAS) utilising a 100-point scale between “Not at all” and “Extremely” with a moveable cursor, were used for appetite (fullness, hunger, thirst, desire to eat, bloated, nauseous, empty), pleasantness and desire to eat, enjoyment and satisfaction ratings. Wording of the instructions for appetite ratings is reported in Appendix A. Pleasantness and desire to eat ratings were given following one mouthful (two pieces of macaroni) before and after the meal (referred to as “Taste tests”; wording of instructions given in the Appendix B in Table A2, as per [28]). Satisfaction may be considered to be a composite of both enjoyment (how tasty the meal was) and post-meal fullness [29] and was included with the following instructions (“How much did you enjoy eating the meal?”; “How satisfied did you feel by the meal?”).

PYY and ghrelin were measured at baseline and every 30 min for 3 h. In addition, blood glucose was measured at baseline and 120 min post-meal to ensure that none of the participants showed insulin resistance or impaired glucose tolerance. The samples were collected via a peripheral intravenous cannula. Blood samples for ghrelin and PYY were collected into aprotinin containing EDTA tubes, inverted and centrifuged in 4 °C at 2500 rpm for 15 min. 1N hydrochloric acid (HCl) and phenylmethylsulfonyl fluoride (PMSF) were added as preservatives. Plasma samples were kept in −80 °C until assayed. Details of the measurement assays can be found in Appendix A.

**Figure 1:**
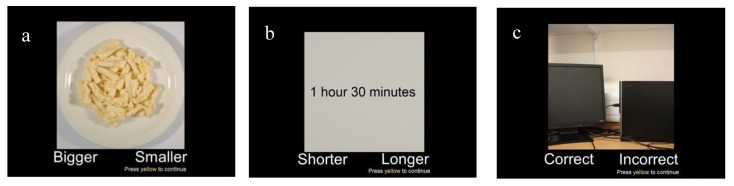
Example screenshots from memory for recent eating task—(**a**) portion size memory trials, in which participants indicated whether the portion on the screen was bigger or smaller than the portion they ate for the meal (**b**) interval since last ate trials, in which participants indicated whether the time on the screen was a shorter or longer time period than when they finished eating (**c**) spatial memory trials, in which participants indicated whether the image on the screen was in the correct or incorrect (mirror image) spatial layout of the environment in which the meal was consumed.

**Figure A1:**
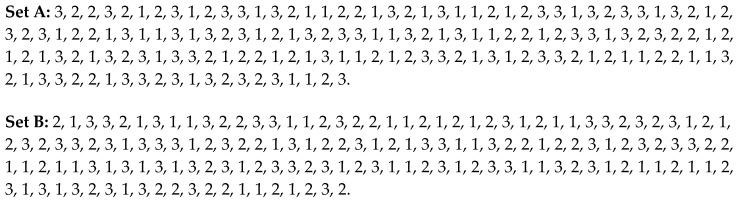
Trial order sets for fMRI task. The numbers represent one of the three trial types (1 = portion; 2 = interval; 3 = spatial).

A novel memory task was developed for this study to examine and distinguish between different aspects of an eating episode that might contribute to an individual’s memory for recent eating, namely, memory for the portion size consumed, the time since eating that portion, and spatial context in which the meal was consumed. This task was designed to measure the behavioural (% correct responses and reaction times) and neural correlates of each trial type. The paradigm was based on an episodic “What, where, when” fMRI task by Kwok et al. [17], in which short video clips were presented, followed by images of the scenes in three different trial types that required recall of *what* was in the video (scene recognition), *when* in the sequence of the clip was the image shown (scene chronology), and *where* items were arranged (correct spatial layout). In our newly adapted paradigm, for each of the three trial types, an image was shown in the centre of the screen. The participant was instructed by an on-screen cue to make a response based on three particular trial types; (a) portion size (b) interval since last ate and (c) spatial memory trials (see Figure 1 for details of trial-types). A total of 150 trials, 50 trials of each type, were pseudo-randomly displayed, in one of two orders (counterbalanced between participants) with no more than two of any one trial type in a row (Figure A1, Appendix A). The 50 portion photographs used in the portion trial tasks were in 20 kcal equicaloric steps either side of the true portion size of the test meal used in the study (600 kcal). Each trial was presented for 4 s, with a mean ISI of 5.5 s (random jitter of between 3–8 s of fixation cross, no more than two of any length in a row). The data captured when the fixation cross appeared was used as the baseline fMRI measure (see Section 2.6 below for details). Response mapping to left/right button presses were counterbalanced between participants. Behavioural performance on the task was measured by percentage of correct responses and response times (RT) for each trial type.

**Figure 2:**
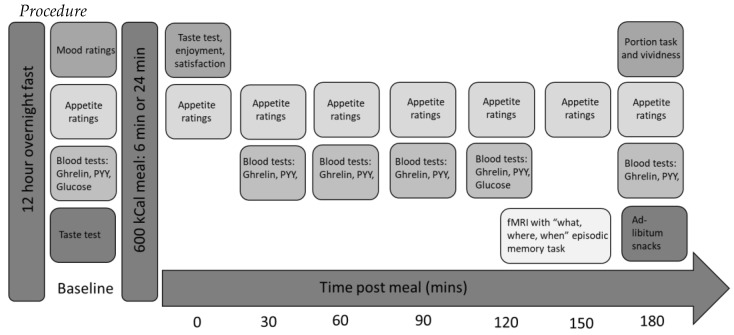
Study design.

Prior to the study session, participants had been advised that the aim of the study was to assess physiological responses to a meal. The main aim of studying the effect of eating rate was partially disclosed from participants. The study session took place at CRIC Bristol utilising a novel protocol (Figure 2). Participants were asked to abstain from eating or consuming calorie-containing beverages for 12 h overnight before the session. On arrival, height (cm) was measured with shoes removed using a stadiometer and weight (kg) was measured using electronic scales. Participants were asked to complete baseline appetite and mood ratings using a VAS on the computer. A cannula was then inserted and a baseline blood sample taken (for glucose, PYY and ghrelin).

Participants were then asked to consume a 600 kcal meal of macaroni cheese (Tesco^®^ Italian Kitchen Macaroni Cheese Pasta, 1.78 kcal/g) in either 6 min (normal rate group) or 24 min (slow rate group) by eating a mouthful, of specified size, cued by an audible bleep. The normal rate group consumed a larger bite size (2 pieces vs. 1 piece of macaroni) with a shorter inter-bite interval (12 s vs. 24 s). A larger portion (562 g, 1000 kcal) was presented to participants such that they never saw the exact portion size consumed. The exact timing of each eating rate group was designed to ensure that only 600 kcal was consumed. This was to test memory of the meal actually consumed rather than relying on recall of the portion size seen on the plate (N.B. pilot work prior to this study showed that presenting participants with the exact 600 kcal portion led to ceiling effects on the novel memory task). To calculate the length of the meals in each condition, a separate group of eight participants (same inclusion criteria) were asked to consume the meal without time constraints (mean time = 6 min). The length of the slow meal was then extrapolated from this mean. Participants were provided with a 250 mL glass of water with their meal, and if they requested extra water they were provided with a further 250 mL glass. Participants then completed the pleasantness and their desire to eat taste tests (pre and post meal), and enjoyment and satisfaction ratings (post-meal only). Appetite ratings and blood samples for measurement of PYY and ghrelin concentrations were collected half-hourly for 3 h post-meal, along with a second glucose measurement at 120 min post-meal.

An fMRI scan was performed 2 h post-meal. Neuroimaging took place at CRIC Bristol on a Siemens 3T Magnetom Skyra MRI scanner using a 32-channel head coil. Participants were supine with cushions around the head in the coil to restrict movement. A pulse-oximeter and respiratory bellows were used to measure heart rate and respiration. Details of the acquisition can be found in Appendix A. During this scan, the novel memory for recent eating task was performed (details above). After the scan, following final blood collection and appetite ratings, participants were asked to report how vividly they remembered consuming the meal using a VAS (“How vividly do you remember the lunch you ate earlier today?”), and were asked to estimate the size of the portion they had consumed using computerised images which each differed by 20 kcal (similar to the ideal portion size task described in [30]). Finally, participants were offered an *ad-libitum* snack 3 h post-meal consisting of 500 kcal of cookies and 500 kcal of crisps, and were advised to “eat until comfortably full”. A further 250 mL glass of water was provided with this snack meal. At the end of the study session, participants were asked to complete a free text awareness question (“Please describe in as much detail as possible what you believe the aim of this study was”).

### 2.5. Behavioural Data Analysis

Statistical analyses were performed using SPSS (IBM SPSS Statistics 23, Armonk, NY, USA). *A priori*, we decided to report effect sizes (Cohen’s d) due to the sample size of this feasibility study. Reporting effect sizes as a statistical approach is advocated by numerous authors because of concerns about null-hypothesis significance testing and because *p* values do not indicate the magnitude of any reported effects [31,32,33]. Confidence intervals for the effect sizes, calculated using SPSS [34], were included to allow interpretation at the population level, as recommended for feasibility studies [35]. For comparisons between the slow rate (experimental) group and normal rate (control) group we have reported standard error and effect sizes, using the Cohen’s d formula as below:

(1) $$\mathrm{Effect}\;\mathrm{size}=\frac{\left(\mathrm{mean}\;\mathrm{of}\;\mathrm{slow}\;\mathrm{rate}\;\mathrm{group}\right)-\left(\mathrm{mean}\;\mathrm{of}\;\mathrm{normal}\;\mathrm{rate}\;\mathrm{group}\right)}{\mathrm{Pooled}\;\mathrm{standard}\;\mathrm{deviation}}$$

Descriptors of magnitude of effect size were 0.20 for small effect size, 0.50 for medium effect size and 0.80 for large effect size [36]. For correlations, Pearson’s correlation coefficient was used and the following descriptors were applied for the magnitude of the correlation coefficient with *r* = 0.10 for small effect size, *r* = 0.30 for medium effect size and *r* = 0.50 for large effect size. Appetite ratings are reported as *change* from pre-meal baseline to each time point (e.g., 120 min rating—pre-meal baseline). These were calculated on an individual participant basis, by subtracting the participant’s baseline score from each of the later time points.

### 2.6. FMRI Data Analysis

Details of the image pre-processing can be found in Appendix A. Time-series statistical analysis was carried out using FMRIBs Improved Linear Model (FILM) with local auto-correlation correction (pre-whitening) [37]. Explanatory variables (EVs) were added to the general linear model for each trial type, with correctly answered trials and incorrect trials as separate regressors. Incorrect trials were not modelled further in the analysis (proportion incorrect portion trials: normal 32%; slow 21%; interval trials: normal 29%; slow 27%; spatial trials: normal 39%; slow 42%). Trials were modelled in two ways (within a single design) by including EVs that accounted for portion, interval and spatial trials versus baseline (a fixation cross) with a weighting of one, and a second set of EVs where the weighting reflected the time taken to respond to each trial (regressors specified onset, duration and RT for each trial type). This second set of regressors determined brain areas whose response was linearly related to trial RT—and constitutes a parametric analysis [38]. These EVs provided additional understanding of the processes underlying the three trial-types as the mean RT to the three trial-types were different. The remaining six EVs modelled the incorrect trials in the same format as the correct trials (weighting of one or with RT). Contrasts were defined to examine the response to each trial type modelled via the different EVs, these contrast of parameter estimates (COPEs) were subsequently used to perform second-level group analyses using two approaches. The first approach was a mixed effect analysis in FEAT using FLAME (FMRIB’s Local Analysis of Mixed Effects stage 1) (details of registration in Appendix A). For each COPE, this analysis estimated the whole sample mean, and unpaired *t*-tests were conducted to estimate differences between normal and slow rate groups. Due to the risk of false positives when analysing imaging data (when analyses are conducted on over 100,000 voxels in the brain), some thresholding is necessary. The conservative approach taken here follows the recent discussion about cluster-based thresholding [39] and employed a cluster threshold of *z* = 3.09, with a corrected *p* value of 0.05. Reporting of cluster location used the FSL tool (Oxford, UK) ATLASQUERY and associated script, AUTOAQ, based on three atlases in FSL: Cerebellar Atlas in MNI152 space after normalization with FNIRT; Harvard-Oxford Cortical Structural Atlas; Harvard-Oxford Subcortical Structural Atlas. The closest to estimates of effect size in fMRI data is to extract the percentage BOLD signal change in the regions of interest and plot the values for each group. Note that the corresponding results for parametric analyses reflect changes in the magnitude of the slope between BOLD and RT.

The second approach was a masked analysis using RANDOMISE, FSL’s tool for nonparametric permutation inference on neuroimaging data [40]. Based on previous literature we defined a priori anatomical regions of interest (ROI) for further analysis. The masks comprised two “networks”: (i) food-related brain regions known from previous literature to respond to satiation and food cues, including hypothalamus, amygdala, nucleus accumbens, striatum, insula and orbitofrontal cortex [19,20,22,23,41]; (ii) memory-related regions known from previous literature to respond to episodic memory, including hippocampus, parahippocampal gyrus, angular gyrus, frontal pole and precuneus cortex [17,18,42]. Masks were created by thresholding the corresponding anatomically defined brain areas in the Harvard-Oxford Cortical and Subcortical structural atlases in FSLview, except the hypothalamus mask that was drawn by hand using the Atlas of the Human Brain [43] as a guide.

The RANDOMISE analysis uses the COPEs for each trial type taken from the first level analyses and transformed into standard space (details in Appendix A). Unpaired t-tests were conducted between the normal and slow rate groups for the response in each of the chosen masks separately, and significance determined using threshold-free cluster enhancement (TFCE) [44], and a FWE-corrected value of *p* < 0.05. Clusters of more than 10 voxels were first reported without accounting for the number of ROIs tested (11). In order to address this potential limitation, we performed additional masked analyses combining all the masks for (i) the food-related regions, (ii) the memory-related regions, and (iii) all masks combined. Unpaired *t*-tests were conducted between the normal and slow rate groups for the response in each of these three combined masks, as above.

## 3. Results

### 3.1. Feasibility Outcomes

#### 3.1.1. Combination of Novel and Technically Challenging Measures

It was possible to collect full data sets from the majority of participants, combining a range of novel and technically challenging measures. Due to unforeseen staff circumstances, one of the study participant’s study sessions was terminated part way through and therefore a further participant was recruited (with a total of 11 participants in the normal rate group and 10 participants in the slow rate group). Specifics details of data points collected for each measure are as follows: 100% (21/21) of data points collected for ratings of baseline appetite, enjoyment, satisfaction, pre and post meal pleasantness and desire to eat, appetite ratings at 0 and 30 min post meal, and ghrelin and PYY levels at 0 and 30 min post-meal. 95% (20/21) of data points were collected for the novel memory for recent eating task in the scanner, memory vividness VAS, portion size task, and post-meal appetite ratings from 60 min time-point onwards. 91% (19/21) of data points were collected for glucose levels at 0 and 120 post-meal, ghrelin and PYY levels at 90, 120 and 180 min post-meal, and ad libitum consumption of crisps and cookies. Finally, 86% (18/21) data points were collected for ghrelin and PYY levels at 60 min post-meal.

#### 3.1.2. Novel fMRI Task

The novel fMRI task was successful; participants were able to perform the task and individual participant scores were neither at floor or ceiling values (i.e., no participant scored the minimum or the maximum score). As a result, it was possible to demonstrate differences between the two groups and between different task types, which is promising for a future larger study.

#### 3.1.3. Regions of Interest Using fMRI

Ability to measure imaging signal in the brain regions of interest was investigated through examination of the first level maps for each participant. These showed that signal change was observed in the regions of interest in the brain.

#### 3.1.4. Blood Sampling Protocol

It was possible to cannulate all study participants on the first attempt. 115/126 (91.4%) blood samples were successfully obtained, and excluding the participant whose study session was terminated early due to external circumstances 113/120 (94.2%) of samples were obtained. Therefore, these young, healthy adult volunteers were willing to have blood taken for research purposes.

#### 3.1.5. Participants Awareness of the Aim of the Study

None of the study participants guessed that the aim of the study was related to eating rate and therefore the actual aim of the study was concealed successfully; removing a potential source of bias.

### 3.2. Preliminary Results

#### 3.2.1. Baseline Characteristics

Following the randomisation procedure, the groups were well matched for each of the variables (Table 1). Baseline appetite ratings are reported in the Appendix B (Table A1).

#### 3.2.2. Post-Meal Outcome Measures

Pleasantness and desire to eat ratings performed after one mouthful of the macaroni cheese (taste test), both before and after the meal are shown in the Appendix B (Table A2). The participants in the slow rate group consumed more water (342 mL; S.D 126) than the normal rate group (257 mL; S.D 156) with a moderate effect size (0.6) between the two groups. The normal rate group reported enjoying the meal (57.5; S.D 21.6) more than the slow rate group (46.6; S.D 24.5) with an effect size of 0.5 (lower CI = −0.4; upper CI = 1.3). The normal rate group also reported higher satisfaction levels immediately post-meal (62.9.0; S.D 13.6) compared to the slow rate group (49.4; S.D 28.2) with an effect size of 0.6 (lower CI = −0.3; upper CI = 1.5).

#### 3.2.3. Memory for Recent Eating Task

The slow rate group achieved a higher percentage of correct responses (effect size 0.4) and responded more quickly (effect size 0.4) than the normal rate group on the portion size trials (Table 2). There were only small differences between the two groups in their performance in the spatial trials (effect size 0.2) and negligible differences in the interval trials (effect size 0.1). Response times to the spatial trials were longer than for the other two trial types.

#### 3.2.4. Portion Task and Vividness

Participants in the slow rate group reported remembering the meal slightly more vividly (71.3; SD 8.0) compared to the normal rate group (64.9; SD 22.5), with a moderate effect size of 0.4 (lower CI = −0.5; upper CI = 1.3). In the portion size task, participants in both the normal (−55.3 kcal; SD 156.4) and slow (−44.0 kcal; SD 131.6) groups remembered having consumed less food than they actually had (effect size = 0.03; lower CI = −0.8; upper CI = 0.9).

#### 3.2.5. Post-Meal Appetite Ratings

**Figure 3:**
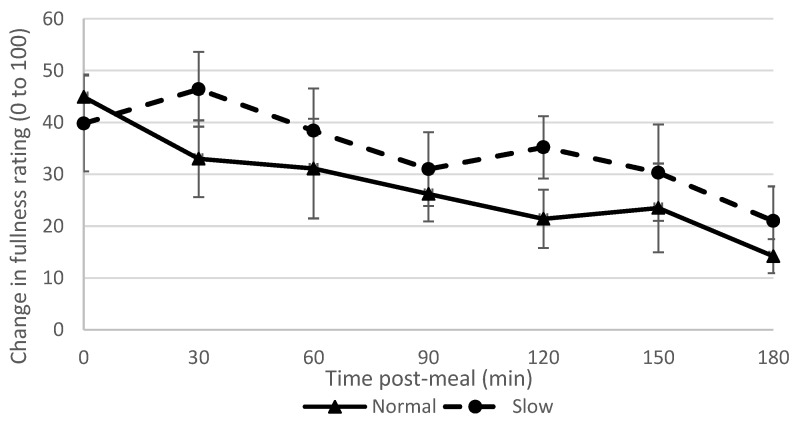
Fullness ratings over time in normal and slow rate groups (error bars = S.E of the mean).

Immediately post-meal, the normal rate group reported a slightly greater change in fullness (44.9; SD 14.5) than the slow rate group (39.8; SD 29.2), with a small effect size (0.2). At 120 min post meal, the slow rate group reported a greater change in fullness (35.2; SD 19.0) than the normal rate group (21.4; SD 17.8) with a large effect size (0.7), as shown in Figure 3. There were small or no differences in effect sizes between groups for the other appetite variables, except for nausea for which the normal rate group reported a greater increase in nausea post-meal than the slow rate group (effect size = 0.7) (Appendix B, Table A3).

#### 3.2.6. Ad Libitum Consumption

The normal rate group consumed both more cookies and crisps at the ad libitum meal, consuming on average 144 kcal more (Table 3).

#### 3.2.7. Blood Results

**Figure 4:**
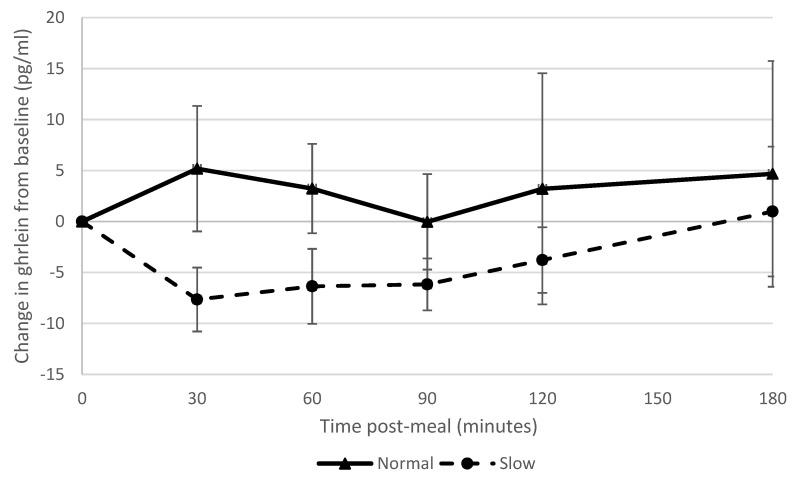
Ghrelin suppression over time in normal and slow rate groups.

**Figure 5:**
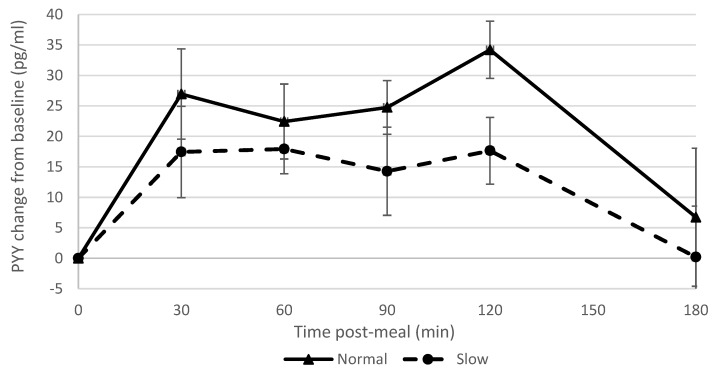
PYY change over time in normal and slow rate groups.

Glucose values were within the normal range both pre- and post-meal for all participants (Table A6). Ghrelin suppression was greater in the slow rate group than the normal rate group, with large effect sizes at 60 and 120 min post-meal (effect size = 0.8; Figure 4, Table A4). Ghrelin levels at 180 min were correlated with ad-libitum intake (*r* = 0.59). PYY levels increased more from the baseline in the normal rate group than the slow rate group, with moderate effect sizes at 30 and 90 min (effect size = 0.6). (Figure 5, Table A4). 

#### 3.2.8. Neuroimaging Results

Whole-brain group analysis examined the areas associated with each trial type compared to rest in the whole sample (*n* = 20, due to failure of imaging data acquisition in a single subject), and for group differences (see Appendix B, Table A5).

Memory for portion size was associated with increased BOLD signal in temporal, parietal and occipital cortices, as well as in the putamen. The normal rate group had a greater response in cuneal cortex (part of the visual system) compared to the slow rate group. Memory for time since eating (interval trials) was associated with increased BOLD response in the lingual gyrus, intracalcarine cortex, occipital pole and putamen compared to rest, with no group differences to report. Spatial memory was associated with an increased BOLD response in the occipital fusiform gyrus, extending into the superior parietal lobule and occipital cortex, with no group differences to report.

When response time (RT) was used to model brain activity (parametric regressors) across the group, a linear relationship between RT and BOLD signal was found in the middle frontal gyrus during portion size trials (Appendix B, Table A5). When comparing groups, a cluster in supramarginal gyrus (parietal cortex) showed a greater response in the slow compared to the normal rate group during the memory for portion size trials. Across the group during spatial trials, the inferior frontal and middle frontal gyri showed a linear relationship between RT and BOLD signal, with no differences between groups. There were no clusters to report for interval trials.

**Figure 6:**
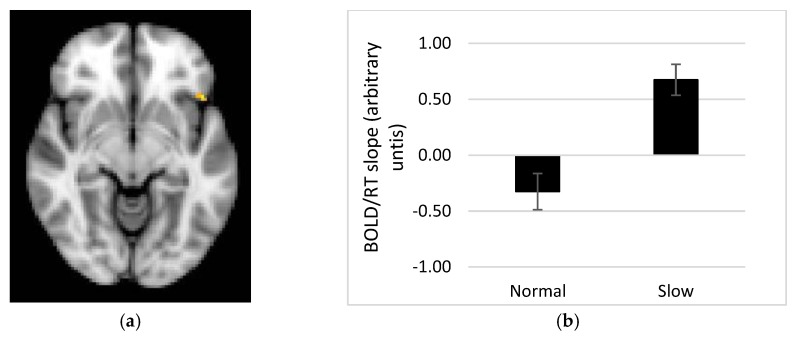
(**a**) Cluster of voxels in the OFC showing a stronger relationship between the BOLD response in this region shown and response times for portion size trials in the slow rate group compared to the normal rate group. This region may show a strong association with memory for eating as linked to task performance. (**b**) Mean % BOLD signal change for the analysis extracted for this region of the OFC for each group (error bars show S.E. mean). The slow group show a steeper BOLD/RT relationship in the region shown in (**a**).

The masked analysis showed no difference between the normal and slow rate groups in the BOLD response for the main effect (modelled with simple EVs) for the portion size, spatial or interval trials. However, when using parametric regressors, differences between groups were found for the portion trials in several masks, see Table 4. These regions all show the same pattern, whereby the slope of the BOLD/RT relationship was steeper in the slow than the normal rate group (example shown in Figure 6). For spatial trials with RT, the nucleus accumbens showed a greater response in the slow rate group compared to the normal rate group. There were no clusters to report for interval trials. No differences were seen in hypothalamus, hippocampus, parahippocampal gyrus, or frontal pole in any of the contrasts.

When the combined masks were analysed, clusters in the OFC (*t* = 5.6, *x* = −46, *y* = 22, *z* = −8), putamen (*t* = 4.38, *x* = 30, *y* = −18, *z* = 6), and insula (*t* = 5.26, *x* = 38, *y* = −4, *z* = 12) survived correction for the portion trials with response time modelled using the “food network” mask in the comparison between the slow and normal eating rate groups. No clusters survived the analysis of the combined “memory network” mask. With all masks combined, a cluster in the OFC remained (*t* = 5.6, *x* = −46, *y* = 22, *z* = −8; Figure 6).

## 4. Discussion

In this study, we sought to assess the feasibility of our novel paradigm that comprised unique and technical challenging measures. Our objectives addressed the feasibility of several aspects of the paradigm; all of which were shown to have positive outcomes, and therefore can be used to guide the design of a future, fully powered study. Moreover, this paradigm was designed to gain a greater understanding of mechanisms underlying the effect of eating rate on energy intake, both cognitive and physiological. This combination of measures, tested in this feasibility study, provided preliminary data to show that slower eating led to a greater feeling of fullness, increased ghrelin suppression and a more vivid and accurate memory of the meal, yet in the context of reduced enjoyment and satisfaction from that meal. Importantly, these effects were associated with a 25% reduction in *ad-libitum* intake for those who ate their previous fixed meal more slowly. This corroborates one previous study with a similar design [11], but is contrary to other studies of similar design [7,8,45], which may in part be due to differing percentage manipulation of eating rate. This is, therefore a relatively novel effect, as many of the previous studies in this field have measured the effect of eating rate within a single meal or by comparing different food textures [3,46,47,48].

In terms of feasibility, the study protocol was acceptable for participants and none of the participants guessed the actual aim of the study, therefore successfully avoiding a potential source of bias. It proved feasible to combine (i) behavioural measures, such as consuming a test meal, and regular appetite ratings, with (ii) physiological measures, such as gastrointestinal hormones and fMRI, in addition to (ii) cognitive measures, such as the memory for recent eating task. The blood sampling protocol was acceptable to participants and demonstrated that young, adult, healthy volunteers were prepared to have blood samples taken for research. The two conditions (normal and slow eating rates) for consuming the meal were acceptable to participants and all participants finished the test meal within the allocated time. The novel ‘what, where, when’ memory paradigm utilised to evaluate memory for recent eating was understood by participants and responses were not at ceiling. Performance on the “what” and “when” memory trials were similar, with a relative reduced accuracy and longer response times on the ‘where’ memory trials. Trial types may not have been well matched in terms of difficulty or spatial memory for a meal may not be as relevant as ‘what’ and ‘when’ in relation to memory for a recent eating episode. These issues will be taken forward into developing the task for future studies.

There are a number of potential limitations of this feasibility study that should be considered in the design of future, fully powered studies. First, we acknowledge that the small sample size renders the effect size estimations imprecise and therefore we have been cautious in our interpretation of the preliminary results. This will be borne in mind when calculating the sample size for future larger studies. *A priori*, it was decided not to apply null hypothesis significance testing (NHST) to this data set. While currently this may be unconventional in this field, this study was designed with feasibility objectives in mind and therefore, in line with CONSORT guidelines for pilot and feasibility studies [49], it would not be valid to perform NHST on the initial data due to the sample size. Secondly, combining the memory and physiological measures was not without difficulties. A between-subjects design was employed (as per [10]) to enable memory for recent eating measures to be included and to avoid possible carry over effects of manipulating the meal within-subjects. However, this design may have been disadvantageous for the hormonal measures, which can vary between individuals. Importantly, all factors were measured relative to an individual’s baseline to account for within-session variability. Careful design considerations are necessary to overcome complications of including cognitive and physiological measures in one design.

Based on our feasibility assessment above, therefore, the recommendations for a future design would be to first perform a series of power calculations for the main outcome measures to gauge the level of sensitivity required for each measure and therefore to ascertain the number of participants required to fully-power those measures. An example power calculation for two brain regions of interest is provided in Appendix B. The challenge for future studies combining diverse measures will lie in powering the whole study to the measure requiring the most sensitivity. The memory task would benefit from some improvements, for example by developing “spatial” trials which enabled a similar performance to the “what” and “when” trials. We would also recommend consideration as to whether to employ the same between subject design as this feasibility study, or to utilise a within subject design to enable potentially more accurate comparison of the two eating rates, whilst minimising the risk of carry over effects.

The slow rate group reported feeling fuller from 30 min post-meal and persisted for the full study duration of three hours. This supports our hypothesis and findings of previous studies [6,10,50,51], and provides evidence that was previously lacking regarding the effect of eating rate in a fixed meal on fullness ratings [3]. Although the slow rate group consumed more water than the normal rate group, previous research has shown that this does not affect fullness or later ad-libitum consumption [52]; however this effect cannot be ruled out [46].

The normal rate group reported enjoying and feeling more satisfied by the meal, again supporting our hypothesis and replicating previous findings [5,11]. This may explain in part why people consume more if they eat more quickly, as it is a more enjoyable activity. This is an interesting finding however, because it could be postulated that savouring one’s food rather than eating it more quickly might be expected to increase enjoyment and satisfaction. Participants in the slow rate group in the study may have found it frustrating being instructed to consume their food more slowly, and that undermined enjoyment, including the enjoyment component of satisfaction. It is possible that there may be an optimal eating rate for individuals, encompassed within a window of tolerance to change; and reducing eating rate beyond that, reduces enjoyment and satisfaction from the meal (as seen here with this experimentally slow condition) which was also suggested by a previous study [53]. Exploiting the boundaries of this tolerance window may be important for the design of eating rate interventions. On the other hand, tolerance to a slower eating rate might increase with repeated experience, so that slower eating becomes the norm. This would worthy of investigation in future studies.

Slowing eating rate showed a large effect on ghrelin suppression, which supported our hypothesis and corroborated the findings of a previous study [9]; however, other studies found no effect of manipulating eating rate on ghrelin suppression [6,7]. There was a strong correlation between blood ghrelin concentrations post-meal and ad libitum snack consumption. Accordingly, reduced appetite stimulation by ghrelin may in part explain why participants who had eaten more slowly consumed fewer snacks. This is also consistent with the finding that participants who had consumed the meal more slowly reported feeling fuller subsequently (i.e., ghrelin is effectively a signal for an empty stomach). The normal rate group showed a greater PYY response than the slow rate group which was in contrast to what we had hypothesised and findings of previous studies [6,7,9] but in agreement with another study [8]. These differences may in part be due to different methods used to manipulate eating rate [8], different percentage alteration in eating rate and different hormone assays [3], and this is clearly an area where further research is required.

At 3 h post meal, the slow rate group reported that they remembered the meal more vividly than the normal rate group. This finding was in contrast to previous studies which found no effect of eating rate on vividness [10,11], although the latter study showed that vividness of the memory of a meal was negatively associated with subsequent ad-libitum intake [11]. Through the novel memory for recent eating task, the slow rate group demonstrated more accurate memory for portion size, and responded more quickly to the portion trials compared to the normal rate group; the latter of which has been previously associated with participants being more confident in their answers and relying on memory rather than guessing [54,55].

Our fMRI results demonstrate a linear relationship between the BOLD response and time to respond to portion size trials in several brain regions, whereby a steeper BOLD vs. RT relationship in the slow rate group might be linked to successful performance on the task. One alternative interpretation of this finding is that improved performance on the memory for recent eating task is associated with reward accompanying the sense of answering the trial correctly, and this might be reflected in activity within reward related brain regions e.g., OFC, amygdala. However, this explanation sits less well with activity in parietal cortex, which showed the same pattern. Memory for portion sizes recently consumed may therefore require recruitment of areas such as the OFC, insula and putamen, as well as precuneus, angular gyrus, supramarginal gyrus and middle frontal and temporal gyri. Those regions showing a direct relationship with response time have a stronger association with the memory processes recruited when recalling recent eating episodes. These regions are in keeping with the areas sub-serving the object recognition task of Kwok et al. [17] (closest equivalent to the portion size task in this study), but not in keeping with the hippocampal activity found associated with the object task in Cheke et al. [18]. The lack of hippocampal activity in response to the current memory for recent eating task maybe due to the lack of integration required [18], as memory elements (portion size, interval and spatial aspects) were studied separately (as per design in [17]).

The fMRI data also reveals brain regions subserving memory for the spatial environment, or context, in which the meal was consumed. Superior parietal and fusiform cortices were associated with the spatial trials, which is in keeping with a similar spatial task in Kwok et al. [15]. Across both groups, a linear relationship between BOLD signal and response time for spatial trials was observed in the inferior and middle frontal cortices, suggesting that these regions are implicated in memory of this kind.

In terms of future applications of this work, investigating whether or not these effects of experimentally manipulating eating rate are also found in childhood would be beneficial, by repeating this study in both normal weight and obese children, ideally with known genetic variability [56]. In view of alternative studies [6,7] that demonstrated responses for other satiety hormones (such as glucagon-like peptide 1 and cholecystokinin), it would be informative to incorporate a wider range of GI hormones into a further study to provide a more complete picture of the endocrine consequences of slowing eating rate. In order to apply the findings of this research, further work is needed in order to design effective interventions to manipulate eating rate on a long term basis, through a range of methods such as environmental modifications [57], behavioural training from childhood [58] and modification of food textures [59,60]. Our findings highlight the need to focus on preserving meal enjoyment and meal satisfaction when reducing eating rate in behavioural interventions.

## 5. Conclusions

In conclusion, this study has provided the opportunity to test the feasibility of several aspects of our eating rate paradigm. The design of future studies can now benefit from the recommendations made based on this feasibility study. Moreover, the preliminary data highlights some of the neural and hormonal pathways that may underpin (at least in part) the effect of slower eating rate on reduced appetite and later eating. The slow rate group reported a greater increase in fullness after the meal and demonstrated greater ghrelin suppression. In addition, the slow rate group recalled their meal more vividly and accurately. Our fMRI data provide information about potential underlying neural responses to a slower eating rate and how this might be related to improved memory for the meal. The slow rate group also subsequently consumed 25% fewer snacks at an *ad libitum* meal three hours later. Therefore, this study provides promising data for the role of manipulating eating rate on subsequent consumption. However, our data also highlight a potential difficulty for interventions to slow speed of eating. The slow rate group reported reduced enjoyment and reduced satisfaction after their meal. Thus, calculating and then exploiting an individual’s tolerance to eating rate change may be important for future interventions.

## Figures and Tables

**Table 1:** Baseline characteristics of normal and slow rate groups. (mean; SD).

	Normal Rate Group	Slow Rate Group
**N**	11	10
**Male/Female**	6/5	5/5
**BMI (kg/m^2^)**	21.8 (2.0)	21.4 (1.7)
**Age (years)**	23.4 (4.7)	22.7 (3.3)
**DEBQ restraint**	2.7 (0.5)	2.5 (1.2)

**Table 2:** Percentage correct and response time on the memory for recent eating task (performed in MRI scanner) (mean; SD).

Trial Type		Normal Rate Group	Slow Rate Group	Effect Size (Cohen’s d)	Lower CI of d	Upper CI of d
Portion	% responses	67.6 (29.5)	79.0 (20.8)	0.4	−0.4	1.3
	Response time (ms)	1760 (376)	1631 (356)	0.4	−0.5	1.2
Interval	% responses	70.5 (30)	73.4 (22.7)	0.1	−0.8	1.0
	Response time (ms)	1822 (570)	1814 (373)	0.0	−0.7	0.7
Spatial	% responses	60.7 (5.7)	58.4 (13.3)	0.2	−0.6	1.1
	Response time (ms)	2258 (295)	2196 (419)	0.2	−0.7	1.0

**Table 3:** Ad libitum energy intake (mean; SD).

	Normal Rate Group	Slow Rate Group	Effect Size (Cohen’s d)	Lower CI of d	Upper CI of d
**Cookies Consumed (kcal)**	214 (115)	157 (122)	0.5	−0.4	1.4
**Crisps Consumed (kcal)**	232 (117)	185 (136)	0.4	−0.5	1.3
**Total ad Libitum (kcal)**	445 (215)	341 (240)	0.5	−0.5	1.4

**Table 4:** Results of the masked neuroimaging analysis: size and peak co-ordinates of areas of differential activation between normal and slow rate groups.

Contrast	No. of Voxels	Peak *t* Value	MNI Co-Ordinates of Peak		
			*x*	*y*	*z*
**Portion with RT**					
Normal > Slow					
Slow > Normal:					
Left OFC	32	5.6	−46	22	−8
Left OFC	10	4.06	−28	18	−18
Left Amygdala	37	3.65	−20	−14	−10
Right Insula	12	5.26	38	−4	12
Left Striatum (putamen)	108	4.36	−24	4	8
Right Striatum (putamen)	83	4.38	30	−18	6
Right Precuneus	24	4.05	8	−54	50
Precuneus	20	3.21	0	−50	44
Angular gyrus	119	3.93	46	−54	46
**Spatial with RT**					
Slow > Normal					
Nucleus accumbens	20	3.38	−12	12	−10

NB. No differences survived for simple EVs modelling portion size, interval and spatial trials, or the parametric EVs for Interval with RT or Spatial with RT Normal > Slow.

**Figure A2:**
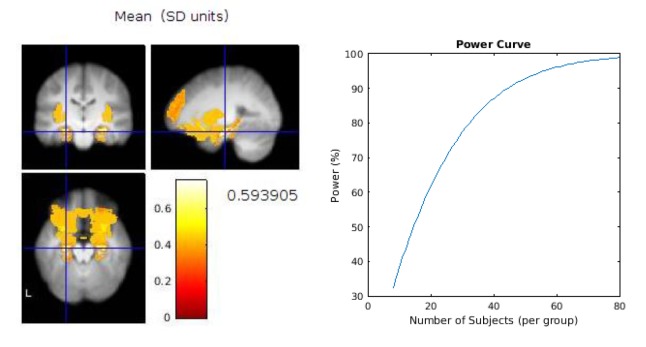
Hippocampus

**Figure A3:**
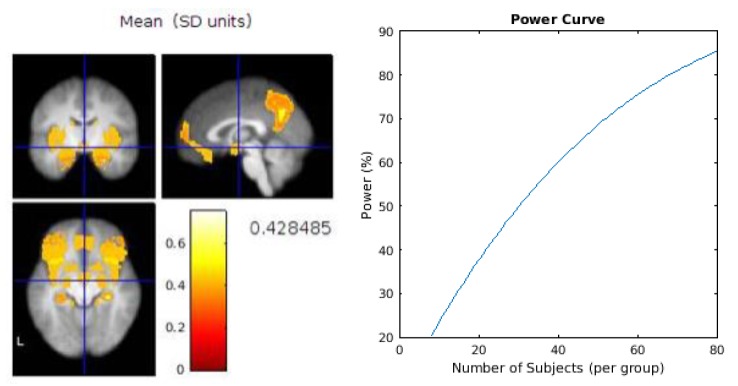
Hypothalamus.

## Appendix A. Additional Methodological Details

### Appendix A.1. Blood Sample Assays

Total active Ghrelin levels were measured by radioimmunoassay (RIA) according to protocol recommendations using a standard curve of known concentration of purified 125I-labeled ghrelin peptide (GHRA-88HK; EMD Millipore Corporation). No plasma dilution was applied when measuring ghrelin levels. The coefficient of variance (CV) for intra-assay variability for was 5.2% and the CV for inter-assay variability was 5.5%.

Total PYY levels were measured by radioimmunoassay (RIA) according to protocol recommendations using a standard curve of known concentration of purified 125I-labeled PYY peptide (PYYT-66HK; EMD Millipore Corporation). No plasma dilution was applied when measuring PYY levels. The coefficient of variance (CV) for intra-assay variability was 3.3% and inter-assay variability was 7.6%.

Blood samples for glucose were collected in sodium citrate bottles and processed the same day using an enzymatic reference method with hexokinase utilising a UV test. The rate of NADPH formation during the reaction is directly proportional to the glucose concentration and is measured photometrically.

### Appendix A.2. fMRI Data Acquisition

Functional MR images were acquired in one run using a multiband sequence [61,62] using the following parameters: TR = 1000 ms, TE = 30 s, Flip angle = 70°, FOV = 192, number of slices = 42 with 20% gap, interleaved, voxel size 2 × 2 × 2.5 mm, multiband acceleration factor = 3, phase encoding = A>>P, GRAPPA acceleration factor 2, bandwidth = 1930 Hz/Px. A maximum of 1800 volumes were sampled over 30 min. Functional data were overlaid on a high-resolution T1-weighted MPRAGE anatomical image for registration into standard space and functional localisation (TR = 2300 ms; TE = 4.2 ms; inversion time = 900 ms; flip angle = 9°; FOV = 224, voxel size = 1 × 1 × 1; 1 mm isotropic voxels).

Pre-processing and statistical analysis of functional images was performed using FMRIBs Expert Analysis Tool (FEAT) [63]. Standard pre-processing steps were followed: motion correction using MCFLIRT [64], non-brain removal using BET [65], spatial smoothing using a Gaussian kernel of FWHM = 5 mm, mean-based intensity normalisation of all volumes, high-pass temporal filtering (cut off 50 s). At first level, the extent of movement by each participant was checked. A movement exclusion criteria of <5 mm over the scan was used; however none of the participants exceeded this. At the first level, the PNM tool in FSL was used to create regressors to model the effects of physiological noise within the general linear model in FEAT [66], temporal derivatives were also included. Registration to high resolution and standard images (MNI “standard” brain, MNI152) was carried out using FMRIB’s Linear Image Registration Tool (FLIRT) [67] and nonlinear warping (FNIRT; 5 mm warp spacing) [68]. Registration was optimised by using field-maps to correct for distortions in the EPI data during pre-processing in FSL [69].

### Appendix A.3. Trial Order for fMRI Task

Figure A1 shows the trial order for fMRI task.

## Appendix B. Additional Results

**Table A1:** Baseline appetite ratings (mean; SD).

	Instruction	Normal Group	Slow Group
Hunger	“I feel hungry”	19.5 (15.3)	12.7 (9.0)
Fullness	“My stomach feels full”	71.3 (13.5)	74.7 (15.6)
Thirst	“I feel thirsty”	57.5 (18.4)	66.7 (13.5)
Desire to eat	“How strong is your desire to eat RIGHT NOW?”	69.1 (16.3)	72.5 (22.7)
Bloated	“I feel bloated”	14.0 (18.4)	23.7 (23.5)
Nauseous	“I feel nauseous”	17.5 (22.2)	39.2 (25.1)
Empty	“I feel empty”	53.8 (21.4)	57.0 (23.1)

**Table A2:** Taste tests.

	Instructions	Normal Group	Slow Group	Effect Size (Cohen’s d)	Lower CI of d	Upper CI of d
**Desire to Eat (pre)**	“Now look at the food in front of you. How strong is your desire to eat, that is, to taste, chew and swallow, the food RIGHT NOW?”	65.7 (19.4)	60.4 (19.4)	0.3	−0.6	1.1
**Desire to eat more (post)**	“How strong is your desire to eat more of the food RIGHT NOW?”	30.5 (28.7)	42.8 (36.9)	0.4	−0.5	1.2
**Desire to eat change**	NA	−35.3 (22.2)	−17.6 (49.9)	0.5	−0.5	1.3
**Pleasantness pre-meal**	“How pleasant does this food taste in your mouth RIGHT NOW?”	79.5 (12.6)	75.9 (2.8)	0.3	−0.5	1.2
**Pleasantness post-meal**	“How pleasant does this food taste in your mouth RIGHT NOW?”	43.7 (6.5)	46.8 (26.2)	0.1	−0.7	1.0
**Pleasantness change**	NA	−35.8 (12.6)	−29.1 (22.7)	0.4	−0.5	1.2

**Table A3:** Changes in mean (s.d.) appetite ratings from baseline immediately pre-meal.

Appetite Rating	Time Post-Meal (mins)	Normal Group	Slow Group	Effect Size (Cohen’s d)	Lower CI of d	Upper CI of d
**Fullness**	0	44.9 (14.5)	39.8 (29.2)	0.2	−0.6	1.1
	30	33.0 (24.6)	46.4 (22.8)	0.6	−0.3	1.4
	60	31.1 (30.4)	38.4 (25.8)	0.3	−0.6	1.1
	90	26.2 (16.7)	31.0 (22.4)	0.2	−0.6	1.1
	120	21.4 (17.8)	35.2 (19.0)	0.7	−0.2	1.6
	150	23.5 (27.1)	30.3 (29.4)	0.2	−0.6	1.1
	180	14.2 (10.4)	21.0 (21.1)	0.4	−0.5	1.3
**Hunger**	0	−46.7 (17.8)	−38.3 (28.8)	0.4	−0.6	1.1
	30	−46.9 (25.4)	−40.4 (26.2)	0.2	−0.6	1.1
	60	−40.3 (24.2)	−39.1 (26.6)	0.0	−0.8	0.9
	90	−34.6 (25.5)	−38.1 (18.5)	0.2	−0.7	1.0
	120	−30.9 (23.4)	−35.8 (19.6)	0.2	−0.7	1.1
	150	−28.9 (18.6)	−30.9 (17.4)	0.1	−0.8	1.0
	180	−18.2 (26.1)	−21.2 (28.1)	0.1	−0.8	1.0
**Thirst**	0	2.7 (21.2)	−19.5 (28.4)	1.0	0.0	1.9
	30	−3.6 (21.8)	−24.8 (30.2)	0.8	−0.1	1.7
	60	−2.2 (15.3)	−24.6 (26.9)	1.0	0.1	1.9
	90	−1.6 (23.0)	−23.6 (24.7)	0.9	−0.1	1.8
	120	2.1 (15.7)	−11.6 (25.2)	0.7	−0.3	1.5
	150	1.2 (16.3)	−4.7 (18.0)	0.3	−0.5	1.2
	180	2.0 (17.6)	−5.4 (23.1)	0.4	−0.5	1.2
**Desire to eat**	0	−50.22 (20.5)	−32.3 (32.2)	0.5	−0.4	1.3
	30	−42.9 (26.0)	−37.8 (28.0)	0.2	−0.7	1.0
	60	−41.1 (19.5)	−42.4 (23.5)	0.1	−0.8	0.9
	90	−36.5 (24.9)	−40.4 (21.1)	0.2	−0.7	1.0
	120	−30.7 (20.5)	−28.6 (22.6)	0.1	−0.8	1.0
	150	−24.5 (16.9)	−28.5 (24.6)	0.2	−0.7	1.1
	180	−16.9 (21.5)	−17.9 (31.8)	0.0	−0.8	0.9
**Bloated**	0	21.6 (25.4)	21.1 (28.3)	0.0	−0.8	0.9
	30	14.0 (18.3)	14.1 (29.4)	0.0	−0.2	0.2
	60	11.8 (16.3)	10.4 (27.8)	0.1	−0.8	0.9
	90	5.9 (20.5)	6.6 (26.8)	0.0	−0.8	0.9
	120	2.5 (18.1)	7.5 (25.3)	0.2	−0.7	1.1
	150	6.2 (25.7)	1.3 (25.9)	0.2	−0.7	1.1
	180	5.5 (13.6)	8.2 (14.5)	0.2	−0.7	1.1
**Nauseous**	0	7.6 (24.8)	−9.8 (21.0)	0.8	−0.2	1.7
	30	1.3 (27.5)	−11.7 (26.1)	0.5	−0.4	1.3
	60	−1.7 (16.8)	−8.3 (24.2)	0.3	−0.6	1.2
	90	−6.1 (16.0)	−12.6 (24.7)	0.3	−0.6	1.2
	120	−5.9 (20.9)	−18.0 (25.0)	0.5	−0.4	1.4
	150	3.5 (29.5)	−15.7 (21.3)	0.7	−0.2	1.6
	180	5.6 (18.6)	−5.3 (17.3)	0.6	−0.3	1.5
**Empty**	0	−36.3 (21.3)	−36.1 (29.7)	0.1	−0.8	1.0
	30	−37.6 (19.4)	−31.0 (33.0)	0.2	−0.6	1.1
	60	−34.6 (18.0)	−31.6 (36.1)	0.1	−0.8	1.0
	90	−31.2 (20.2)	−32.8 (27.8)	0.1	−0.8	0.9
	120	−24.2 (23.6)	−27.9 (28.4)	0.0	−0.7	1.0
	150	−24.6 (18.0)	−23.0 (29.9)	0.1	−0.8	0.9
	180	−9.0 (18.5)	−9.7 (33.1)	0.0	−0.8	0.9

**Table A4:** Change in satiety hormone levels (ghrelin and PYY) from baseline (mean; SD).

	Minutes Post-Meal	Normal Group	Slow Group	Effect Size (Cohen’s d)	Lower CI of d	Upper CI of d
**Ghrelin**	**30**	5.2 (23.1)	−7.7 (10.5)	0.7	−0.2	1.6
	**60**	3.2 (11.9)	−6.4 (11.0)	0.8	−0.1	1.8
	**90**	−0.03 (9.9)	−6.2 (7.6)	0.7	−0.2	1.6
	**120**	3.2 (8.0)	−3.8 (9.7)	0.8	−0.2	1.7
	**180**	4.7 (11.8)	1.0 (19.1)	0.2	−0.7	1.2
**PYY**	**30**	26.9 (19.5)	17.4 (12.8)	0.6	−0.7	1.2
	**60**	22.4 (13.2)	17.9 (21.7)	0.3	−0.7	1.2
	**90**	24.7 (14.8)	14.3 (16.4)	0.7	−0.3	1.6
	**120**	34.2 (35.8)	17.6 (25.1)	0.5	−0.4	1.4
	**180**	6.7 (35.0)	0.2 (45.9)	0.2	−0.7	1.1

**Table A5:** Whole-brain analysis Results.

	Brain Region	Cluster No.	No. of Voxels	Peak *z* Value	MNI Co-Ordinates of Peak		
**Portion trials**					*x*	*y*	*z*
	*Group mean (n = 20)*						
	Occipital Fusiform Gyrus (10.4%)	5	8690	5.07	50	−60	−18
	Precentral Gyrus (19.2%); Postcentral Gyrus (30.3%)	4	970	4.58	−34	−34	46
	Left Putamen (51.6%)	3	277	4.42	−26	−8	6
	Cingulate Gyrus, posterior division (71.4%)	2	189	4.38	0	−30	34
	Superior Parietal Lobule (43.9%)	1	94	3.73	−32	−54	66
	*Normal > Slow*						
	Cuneal cortex (49.0%)	1	236	4.16	−4	−86	32
	*Slow > Normal*	0					
**Interval trials**	*Group mean (n = 20)*						
	Intracalcarine Cortex (10.2%); Lingual Gyrus (14.9%); Occipital Fusiform Gyrus (12.0%)	4	2713	5.26	8	−88	−2
	Precentral Gyrus (18.1%); Postcentral Gyrus (30.1%)	3	1677	4.91	−40	−22	54
	Juxtapositional Lobule Cortex (formerly Supplementary Motor Cortex) (43.0%); Paracingulate Gyrus (17.6%)	2	529	4.14	8	4	58
	Left Putamen (92.0%)	1	109	4.13	−30	−6	2
	*Normal > Slow*	0					
	*Slow > Normal*	0					
**Spatial trials**	*Group mean (n = 20)*						
	Peak in Lateral Occipital Cortex, superior division (15.2%), extends to occipital fusiform gyrus, temporal fusiform cortex, superior parietal lobule, and lingual gyrus	1	10554	5.65	34	−64	−8
	*Normal > Slow*	0					
	*Slow > Normal*	0					
**Portion RT trials**	*Group mean (n = 20)*						
	Middle Frontal Gyrus (23.9%); Precentral Gyrus (13.2%)	1	90	3.82	−32	−2	54
	*Normal > Slow*	0					
	*Slow > Normal*						
	Postcentral Gyrus (41.2%); Supramarginal Gyrus, anterior division (21.4%)	1	105	3.86	52	−28	42
**Interval RT trials**	*Group mean (n = 20)*	0					
	*Normal > Slow*	0					
	*Slow > Normal*	0					
**Spatial RT trials**	*Group mean (n = 20)*						
	Inferior Frontal Gyrus, pars triangularis (19.3%); Inferior Frontal Gyrus, pars opercularis (19.4%)	2	298	3.89	−50	36	2
	Middle frontal gyrus	1	97	3.94	−44	20	26
	*Normal > Slow*	0					
	*Slow > Normal*	0					

**Table A6:** Glucose levels at baseline and 120 min post-meal (mean; SD).

	Time of Sample	Normal Group	Slow Group
**Glucose**	Pre-meal	4.4 (0.2)	4.5 (0.4)
	Post-meal (120 min)	5.3 (1.1)	4.8 (0.6)

### fMRI Power Calculation

To help inform future studies examining similar processes using fMRI, we ran a power analysis based on the methods developed by Mumford and Nichols [70] and implemented in the fMRIpower software package (fmripower.org, Austin, TX, USA). This technique estimates the power (for a range of sample sizes) to detect significant activation within specific regions of interest, with the assumption that the planned studies will have the same number of runs per subject, runs of the same length, similar scanner noise characteristics, and data analysis with a comparable model. The mask used here included: hypothalamus, amygdala, nucleus accumbens, striatum, insula, orbitofrontal cortex, and hippocampus, parahippocampal gyrus, angular gyrus frontal pole and precuneus cortex. The effect sizes have been expressed in standard deviation (sd) units, which is analogous to the Cohens D measure. The reported “power curves” reflect the power to detect a significant difference between two groups with an unpaired *t*-test and corrected *p*-value of *p* < 0.05 within: (D1) the hippocampus and (D2) the hypothalamus. These data reveal that a study performed with approx. 35 subjects would have at least 80% power to detect an effect of size 0.59 sd units in the hippocampus, whereas in the hypothalamus, with approx. 65 participants one would have 80% power to detect an effect of size 0.42 sd units.
